# Ultrastructural Changes in Spinal Motoneurons and Locomotor Functional Study after Sciatic Nerve Repair in Conduit Tube

**Published:** 2012

**Authors:** Hamdollah Delaviz, Abolfazel Faghihi, Jamshid Mohamadi, Amrollah Roozbehi

**Affiliations:** 1 *Cellular and Molecular Research Centre, Department of Anatomy, Faculty of Medicine, Yasouj University of Medical Sciences, Yasouj, Iran*; 2 *Department of Anatomy, Faculty of Medicine, Tehran University of Medical Sciences, Tehran, Iran*; 3 *Department of Physiology, Faculty of Medicine, Yasouj University of Medical Sciences, Yasouj, Iran*

**Keywords:** Motor neuron, Rat, Recovery, Sciatic nerve

## Abstract

**Objective(s):**

*Motor deficit and neuron degeneration is seen after nerve transection. *The aim of this study is to determine whether a poled polyvinelidene fluoride (PVDF) tube with other supportive strategies can protect the *neuronal morphology and motor function after sciatic nerve transaction in rats.*

**Materials and Methods:**

After transection of the left sciatic nerve in 60 male Wistar rats (200-250 g), the epineural group was sutured end to end. In the autograft rats, a 10 mm piece of sciatic nerve was rotated 180 °C and sutured back into the nerve gap. In the nerve guidance channel (NGC) group, polarized piezoelectric PVDF tube containing NGF and collagen gel was sutured in the gap. In control group sciatic nerve was removed (10 mm) without repair. After one, four and eight weeks, the L4-L6 spinal cord segment was removed for histological study using transmission electron microscope. Functional outcome was assessed using the Basso, Bresnahan and Beattie (BBB) locomotor scale at both four and eight weeks after the lesion.

**Results:**

Chromatin condensation was seen after 4 weeks in the repair groups. Cell membrane shrinkage and mitochondrial degeneration was observed after 4 and 8 weeks respectively, in the autografted and NGC rats. In the control group, chromatin condensation, cell membrane shrinkage with mitochondrial degeneration and vacuolization of perikaryon was seen after 1, 4 and 8 weeks, respectively. At 56 days, the functional recovery of the epineural rats significantly increased in comparison to the other groups (*P*< 0.05).

**Conclusion:**

The epineural suture has more efficacies, and NGC may be used as a proper substitute for autograft in nerve injury.

## Introduction

Peripheral nerve injury (PNI) continues to be a major challenge in reconstructive surgery. Permanent disabilities and multiple morphological changes have been seen following nerve transection (1, 2). In the first few days after PNI, Schwann cells proliferate and form bands of Büngner, which provide a guiding conduit for the regrowing axons (3). To regulate this cellular change and to promote locomotor perfomance, external intervention is necessary (4, 5). The current clinical approach for the repair of peripheral nerve defects involves the use of autologous nerve grafts or epineural suture, both of which face many limitations (6, 7). Autograft is usually limited by mismatch of nerve type or size. The long nerve gap and tension at the suture line is the main restriction for the epineural technique (8). A conduit tube is an alternative approach that provides longitudinal support and a diameter that is wide enough to support axonal regeneration (9). In this regard, several strategies have been devised to promote functional recovery after PNI, such as biodegradable polymers (10), bioactive poly (l-lactic) conduits seeded with Schwann cells (11), Permeable conduit containing microfilament scaffolds (12), and collagen tubes (13). 

There is evidence that insertion of expanded polytetrafluoroethylene (ePTFE) tube as a nerve channel in response to a sciatic nerve defect can protect the myelinated axon (14). Polyvinylidene floride (PVDF) and polarized PTFE tubes may provide microenvironment conducive for nerve growth (15). PVDF and PTFE tubes may be combined with other treatments to improve functional recovery following PNI. This study sought to find out whether placement of a polarized PVDF tube, along with the addition of growth factor and collagen gel, enhances locomotor outcome and improves spinal motor neuron restoration after sciatic nerve transection in rats.

## Materials and Methods

***Preparation of polarized piezoelectric PVDF tube***

The polyvinylidene fluoride (Harvard Apparatus Ltd) tube was polarized in the electronic laboratory of Sharif Industrial University as follows: A thin wire was inserted in the lumen of the PVDF tube serving as an inner electrode, while a circumferential array of steel needles served as the outer electrode. The outer needle electrode was connected to the positive output of a voltage supply and the inner electrode was grounded. The voltage output was gradually increased to 21 kv over a 2 hr period and was maintained at that level for 12 hr (15). The tube was then cut into 14 mm pieces; sterilized by 70 % ethanol; filled with 1.28 mg/ml of collagen gel (Roche, Germany) and 100 ng/ml of NGF75 (Roche, Germany); and finally placed in a humidified 37º C incubator for polymerization.

***Animals and surgical procedure***

The experimental protocol was approved by the Tehran Academy of Sciences Review Committee for the use of Animal Subjects (Ethics Local Committee), Tehran. Sixty male Wistar rats (200-250 g) (Pasteur Institute, Tehran, Iran) were assembled into four experimental groups: epineural suture group, autograft group, nerve guidance channel group and a control group. The rats were housed in plastic cages with free access to food and water. Their room was maintained at temperature of 22-24 ^○^C under 12 hr light/ 12 hr dark cycle. Intraperitoneal ketamine (100 mg/kg) plus xylazine (10 mg/kg) was used as a general anesthetic in all surgical procedures. Under aseptic conditions, the skin and muscles of the back of the left thigh were incised, and the sciatic nerve was exposed between the ischial spine and popliteal fossa superior to its bifurcation. 

In the epineural suture group, the left sciatic nerve was transected in the middle of the thigh and then sutured end to end. In the autograft group, a 1-cm segment of the nerve was resected, rotated 180 ^○^C, and sutured back into place, with the proximal and distal nerve stumps being in reverse order. In the nerve guidance channel group (NGC), a 1 cm segment of the nerve was resected. The proximal and distal nerve stumps were then inserted into a 14 mm polarized PVDF tube filled with collagen and NGF and fastened with a single 10-0 epineural suture at its proximal and distal ends. In the control operated animals, one cm of sciatic nerve was removed in the same manner but not repaired.

***Electron microscopic study***

Histological analysis of 5 rats from each group was done 1, 4 and 8 weeks after surgery. Animals were deeply anesthetized and perfused transcardialy by 0.9% heparanized saline, followed by fixation with 4% paraformaldehyde (0.1 M phosphate buffer, pH 7.4). The embedded spinal cord (L4-L6) was dissected out, bathed for 2 hr in 2.5% glutaraldehyde, then dehydrated and washed in 0.1 cacodylate buffer and finally postfixed in 1% osmium tetroxide containing 0.8% potassium ferrocianide and 5 nM calcium chloride in 0.1 M cacodylate buffer for 90 min. After washing, samples were stained with 1% uranyl acetate overnight, dehydrated in graded acetone, infiltrated with Poly/Bed 812 resin (Polysciences, Inc., Washington, PA) and polymerized for 60 hr. Five-hundred-nm-thick sections of the segment were made on an ultramicrotome (Leica ultracut UCT) sections and stained with toluidine blue. Cover slips were added to the sections, and images were taken with the aid of a digital camera (DP 11, Japan) attached to the microscope (Olympus Ax70). Ultra thin sections (50-70 nm) were collected on copper grids for transmission electron microscopy (Ziess, EM 900). Intracytoplasmic vacuoles, pyknotic neuron, increase nuclear condensation and marginal chromatin were detected in the different groups.

 Five grids in different parts of the gray matter in the left anterior horn were prepared in each group. The morphological changes of the nucleus, chromatin condensation, mitochondria, endoplasmic reticulum and cell membrane were studied closely in 13 motor neuron cells from each group. The morphological changes for each change were categorized into four ranges: 1-10% (+); 10-20% (++); more than 30% (+++); and (-) were designated when no morphological change was seen.

***Behavioral assessment***

After transection of the sciatic nerve, five rats from each group were randomly chosen for behavioral assessment. At the end of 4 and 8 weeks after the lesion, hindlimb motor function was assessed based on the Basso, Beattie, and Bresnahan (BBB) Locomotor Rating Scale (16, 17). To conduct a BBB assessment, the rats were allowed to move individually for 5 min on a smooth, nonslip floor in an open field (200 x 100 cm). Hindlimb motor function was scored from 0 to 21 based on the performance of the ipsilateral hindlimb by a trained observer who was oblivious to the identity of the groups.

***Data analysis***

We used SPSS software 7th version to analyze the data descriptively and analytically. Behavioral data was obtained as a mean±SD and analyzed using one-ways ANOVA. *Post* *hoc* comparisons were made using the Tukey’s test. Differences between the groups were considered significant at *P*< 0.05. 

## Results

***Electron microscopy***

**Figure 1:**
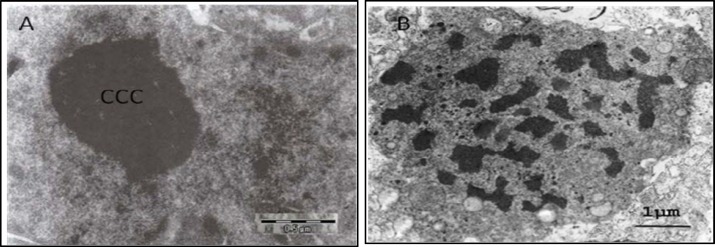
In the control group (A), a pyknotic degenerative nucleus with severe central chromatin condensation (CCC) was seen one week after surgery (X20400). An advanced stage of apoptosis, indicated by chromatin condensation, scattering and clumping, was seen in the nucleus of the epineural suture group (B) 8 weeks after surgery (X10000)

**Figure 2:**
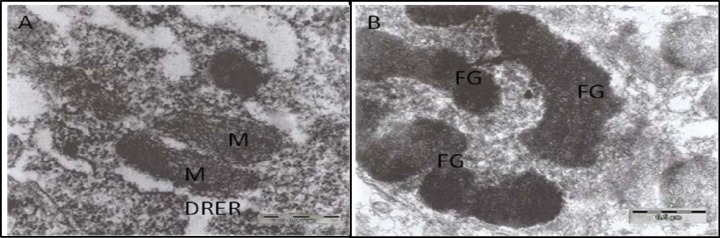
Electron micrographs (A) of the cytoplasm of the motor neuron cell in never guidance channel (NGC) rats show electron dense mitochondria (M) with unidentified cistern and dilated rough endoplasmic reticulum (DRER) after 8 weeks of surgery (X51000). In the neuron of the autograft group (B), more progressive changes were seen, i.e. separated, round to ovoid fragments (FG) of condensed chromatin 8 weeks after surgery (X34000)

One week after surgery there was no morphological changes in the lumbar spinal motor neuron treatment groups, but there was nuclear densities and chromatin condensation in the control group (Figure 1). Ultrastructural changes including central and peripheral chromatin condensation was seen in all rats after 4 weeks. At this time the cell membrane was morphologically normal in the epineural group. In contrast, cell membrane shrinkage was seen in the other groups, as well as mitochondria degeneration in the control group. Cell membrane changes were not seen in the epineural group until after 8 weeks. At week 8, endoplasmic and mitochondria degradation and cytoplasmic swelling were seen in both NGC and autograft groups (Figure 2). In fact, the morphological changes seen in the NGC and autograft groups were very similar to each other throughout the entire 8 weeks (Table 1). 

***Locomotor outcome***

**Figure 3:**
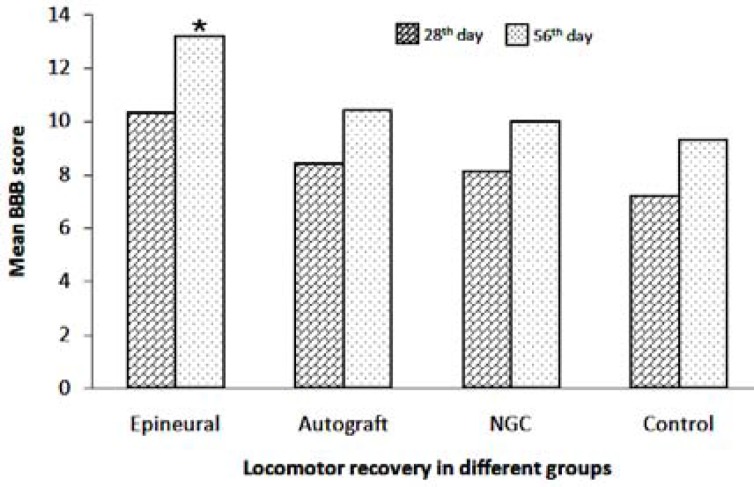
BBB locomotor testing at 28 (left column) and 56 (Right column) days in different groups. At 56 days the epineural rats demonstrated a significant increase in movement of their hindlimbs compared to the other groups (n= 5, *P< 0.05). The autograft and NGC rats received nearly identical BBB scores at both 28 and 56 days post- injury.

Twenty eight days after sciatic nerve transection, the epineural group showed better locomotor performance of plantar placement with no weight support compared to the other groups. The BBB scale measurements were as follows (mean±SD): 10.31±.31, 8.13±1.45, 8.45±.66 and 7.29±.64 for the Epineural, NGC, Autograft and control groups, respectively (Figure 3). One-way ANOVA determined a statistical difference (*P*< 0.05) of functional recovery in the epineural group compare to the other groups at 65 days (Figure 3). At this time the NGC rats demonstrated paw placement with weight bearing, but there was no significant difference compared to the control or autograft groups.

**Table 1:** Ultrastructural change in motorneurons of the left ventral horn at the level of L4-L6 at 1, 4 and 8 weeks after surgery (morphological changes for each group were categorized into four ranges, including 1-10% (+), 10-20% (++), more than 30% (+++)) and no change (-)

Changes		Groups	Central chromatin condensation	Peripheral chromatin condensation	Cell membrane shrinkage	Mitochondria and endoplasmic degeneration	Cytosol vacuolization
Control	1^st^ week		+	+	-	-	-
	4^th^ week		++	++	+	+	-
	8^th^ week		+++	+++	+++	++	++
Epineural suture	1^st^ week		-	-	-	-	-
	4^th^ week		+	+	-	-	-
	8^th^ week		+	+	+	-	-
Autograft	1^st^ week		-	-	-	-	-
	4^th^ week		+	+	+	-	-
	8^th^ week		++	++	+	+	+
NGC	1^st^ week		-	-	-	-	-
	4^th^ week		+	+	+	-	-
	8^th^ week		++	++	+	+	+

## Discussion

Various bioengineered nerve grafts have been developed from polymeric materials that have well-tailored properties and dimensions to meet the requirements for peripheral nerve regeneration. These materials range from naturally derived polymers to conventional nondegradable and biodegradable synthetic polymers. Generally, an ideal nerve guide should be non-cytotoxic, highly permeable, and sufficiently flexible with suitable degradation rate and products to provide guidance for regenerative axons and to minimize swelling and inflammatory responses. This study assessed a poled PVDF tube with growth factor and collagen gel as to whether it can enhance locomotor recovery as an autograft transplant.

Functional recovery failed in the control group in the absence of treatment of the transected nerve. Ultrastrutural findings were confirmed with behavioral assessment. Following peripheral nerve axotomy and block of axonal transport, certain morphological changes occurred in the spinal motor neuron (1). Chromatin clumping and an advanced stage of apoptosis were seen two months following sciatic nerve transection in different groups. This result suggests promoted recuperation is needed to prevent neuronal degeneration after nerve transaction. NGC is a treatment approach that can be made compatible with the size of a nerve defect. Also, it can be manipulated to take full advantage of a restorative constitution (18). In the epineural rat group, the locomotor activity increased and morphological changes seen were less severe compared to the other groups. Our results support literature studies that report the best surgical method for nerve repair is epineural suture by end–to-end approximation of the stumps (17). However, end-to-end approximation is not feasible when there is tension at the lesion site. It is documented that tension at the repair site has an adverse effect on axonal regeneration (19).

Another approach treatment which is extensively used for nerve repair is autograft, but autograft is limited in that only nerves with suitable diameter, size and length can be used. Furthermore, nerve autograft is often accompanied with secondary injury at the donor site, which has led to its decline in use. Our data indicates that when there are large gaps, a guidance channel is needed to bridge the proximal and distal nerve stumps for axonal regeneration. In addition, a polarized piezoelectric polyvinelidene fluoride tube can provide an appropriate microenvironment for long nerve regeneration. It should be noted that the morphologycal changes and locomotor evaluation in the NGC rats was the same in the autograft group after 8 weeks. This result suggests that the PVDF tube along with other strategies could improve nerve regeneration of a long gap, whereas the epineural or autograft could not do so. Besides, additional problems such as hyperesthesia and neuromas did not occur at the PVDF tube donor site (20, 21). In addition, an electrical charge is favorable for nerve regeneration (22), and the PVDF tube creates a transient surface with little strain to help nerve outgrowth (23). The increase of growth factor in the PVDF tube decreases degenerative neuronal morphological changes. Conversely, the lack of these factors may lead to impairment of the neuron structure or loss of the neuron (24). In the present study it may be the neurotrophic factors transported from the tissue organs to the motor neurons that aid the regenerating axon. Other supportive therapies such as growth factor, cellular and molecular treatments can be used alongside the conduit tube (25, 26). There are promising indications of nerve repair seen with a nerve gap less than 15 mm when treated with nerve conduit of poly (l-lactide-co-glycolic acid)-coated collagen tube filled with collagen gel (27). Furthermore, collagens in the extra cellular matrix within the conduit tube act as nerve guide and improve nerve regeneration (28). However, there is a report that the collagen tube bridge collapsed into a nerve gap that was 15 mm long (29). 

The poled PVDF tube with trifluoroethylene (PVDF-TFE) demonstrated more efficient repair of a rat sciatic nerve gap that was longer than 10 mm compared to the unpoled nerve guidance channel (15). In our study, less advanced morphological changes seen in the NGC rat group compared to the control group illustrates that this technique may effectively allow for nerve regeneration, perhaps through a permissive trophic environment. Similarly, piezoelectric guidance channels show improved neuronal regeneration after sciatic nerve transection in mice. However, another study has shown how a poly-tetrafluoroethylene prostheses tube may act as a foreign body and caused an immune reaction by giant cells (18).

## Conclusions

 Polarized piezoelectric PVDF tube is beneficial in directing axonal regrowth from the proximal stump toward the distal end and has the capacity to merge with other beneficial therapies. Thus, in order to develop fully restorative treatment for PNI, further investigation into electrically conducting polymers is necessary. 
